# Vascular smooth muscle cell contraction and relaxation in the isolated aorta: a critical regulator of large artery compliance

**DOI:** 10.14814/phy2.13934

**Published:** 2019-02-27

**Authors:** Arthur J. A. Leloup, Cor E. Van Hove, Sofie De Moudt, Guido R. Y. De Meyer, Gilles W. De Keulenaer, Paul Fransen

**Affiliations:** ^1^ Laboratory of Physiopharmacology Department of Pharmaceutical Sciences University of Antwerp Antwerp Belgium; ^2^ Laboratory of Pharmacology Faculty of Medicine and Health Sciences University of Antwerp Antwerp Belgium

**Keywords:** Aortic stiffness, basal NO, mouse, vascular smooth muscle tone

## Abstract

Over the past few decades, isometric contraction studies of isolated thoracic aorta segments have significantly contributed to our overall understanding of the active, contractile properties of aortic vascular smooth muscle cells (VSMCs) and their cross‐talk with endothelial cells. However, the physiological role of VSMC contraction or relaxation in the healthy aorta and its contribution to the pulse‐smoothening capacity of the aorta is currently unclear. Therefore, we investigated the acute effects of VSMC contraction and relaxation on the isobaric biomechanical properties of healthy mouse aorta. An in‐house developed set‐up was used to measure isobaric stiffness parameters of periodically stretched (10 Hz) aortic segments at an extended pressure range, while pharmacologically modulating VSMC tone and endothelial cell function. We found that the effects of α1‐adrenergic stimulation with phenylephrine on the pressure‐stiffness relationship varied in sensitivity, magnitude and direction, with the basal, unstimulated NO production by the endothelium playing a pivotal role. We also investigated how arterial disease affected this system by using the angiotensin‐II‐treated mouse. Our results show that isobaric stiffness was increased and that the aortic segments demonstrated a reduced capacity for modulating the pressure‐stiffness relationship. This suggests that not only increased isobaric stiffness at normal pressure, but also a reduced capacity of the VSMCs to limit the pressure‐associated increase in aortic stiffness, may contribute to the pathogenesis of this mouse model. Overall, this study provides more insight in how aortic VSMC tone affects the pressure‐dependency of aortic biomechanics at different physiological and pathological conditions.

## Introduction

The isometric contraction of isolated thoracic aorta segments in response to pharmacological or mechanical stimulation has been extensively investigated over the past few decades. Many studies have contributed significantly to our overall understanding of the local, humoral, mechanical, and neurogenic regulation of the molecular pathways involved in thoracic aorta vascular smooth muscle (VSMC) contraction and relaxation (Russell and Watts 2000; Fransen et al. 2015; Leloup et al. 2015). Dysfunction of these pathways has been shown to contribute to numerous pathologies, including hypertension and arterial stiffness (Wilkinson et al. 2002; Lindesay et al. 2010). However, the exact physiological role of VSMC contraction and relaxation in the healthy aorta is presently unclear.

With each heartbeat, the left ventricle ejects blood into the aorta, which acts as an elastic reservoir to store energy when dilating during systole and to release this energy by elastic recoil during diastole. This allows the conversion of the heart's pulsatile flow to a nearly continuous flow in the peripheral capillaries, a phenomenon known as Otto Frank's Windkessel theory. Interestingly, the relationship between the elasticity of the aorta and blood pressure – when normalized for the mean physiological pressure – is remarkably similar in all vertebrates and invertebrates with a closed circulatory system (Shadwick 1999). This strongly suggests that the mechanical design of the aorta is subject to a strong evolutionary pressure and is a crucial determinant of cardiovascular (CV) health.

The functional and biomechanical properties of the more distally located muscular arteries are considerably different from the elastic arteries. They are stiffer, show limited age‐related stiffening, have higher relative VSMC content and regulate blood flow through an active change in diameter (Laurent et al. 1994; Guo and Kassab 2003; Bia et al. 2005; Ruitenbeek et al. 2008; Borlotti et al. 2012; Zhang et al. 2013; Dinardo et al. 2014). Muscular artery VSMCs differ from elastic artery VSMCs in terms of protein expression profiles, morphology, and intrinsic rigidity (Dinardo et al. 2014). Also, elastic arteries – but not muscular arteries – produce large amounts of basal nitric oxide (NO) ex vivo (Leloup et al., 2015) and the overall contribution of NO to basal tone is larger (Lefroy et al. 1993; Shimokawa et al. 1996). Altogether, this indicates that the morphological, biomechanical, molecular, and contractile properties of the VSMCs are adapted to meet physiological requirements, depending on their location in the arterial tree.

Although the relative VSMC content of the thoracic aorta is lower compared to any other part of the arterial tree, VSMCs are still the predominant cell type in the aortic wall and they account for approximately 40% of total wall volume in mice (Dinardo et al. 2014) and canine (Apter et al. 1966) thoracic aorta. VSMCs in large arteries are known to organize and synthetize extracellular matrix proteins (which crucially determine the nonlinear pressure‐stiffness relationship of the aorta) during development and in response to hemodynamic stress or injury (Wagenseil and Mecham 2009). However, the specific contractile design of the aortic VSMCs and the fact that VSMC activation can decrease aortic diameter by 20–50% (Nichols et al. 2011), indicates that VSMC contraction and relaxation functionally contribute to the physiological regulation of large artery compliance. However, even after decades of research into the isometric contraction properties of thoracic aorta rings, their exact physiological role in the intact organism is still unclear.

In this study, we characterized the acute effects of VSMC contraction and relaxation on the isobaric biomechanical properties of the healthy aortic wall. Given the unique ability of the large arteries to produce large amounts of basal NO and the observed link between reduced NO bioavailability and arterial stiffness (Fitch et al. 2001; Soucy et al. 2006; Leloup et al. 2014), we hypothesized that basal NO would be an important physiological regulator of aortic biomechanics. We used the Rodent Oscillatory Tension Set‐up to study Arterial Compliance (ROTSAC), an in‐house‐developed set‐up to assess the stiffness of intact aortic rings at a broad pressure range, in a highly controllable extracellular environment, while stretched at high frequency (10 Hz) to simulate the physiological heart rate in mice. Our findings show that the magnitude, direction, and sensitivity by which VSMC stimulation affected the isobaric properties of the isolated aortic segment strongly depended on the distension pressure and basal NO efficacy. To our knowledge, this is the first report of an experimental model that describes how the physiological properties of the VSMCs – in cross‐talk with the ECs – determine the pressure‐dependency of aortic biomechanics in the isolated thoracic mouse aorta.

## Materials and Methods

### Ethical approval

All animals were housed in the University of Antwerp animal facility in standard cages with 12–12 h light‐dark cycles, with free access to regular chow and tap water. This study was approved by the Ethical Committee of the University of Antwerp and all experiments were performed conform to the Guide for the Care and Use of Laboratory Animals, published by the US National Institutes of Health (NIH Publication No. 85–23, revised 1996).

### Aortic tissue preparation

The following animal groups were used: maximal PE experiments: ~3‐month‐old C57BL/6JRj males (Janvier Labs), mean body weight: 29.0 ± 0.7 g, *n* = 5, PE sensitivity experiments: ~6‐month‐old C57BL/6JRj males (Janvier Labs), mean body weight: 33.0 ± 1.3 g, *n* = 5. Mice were euthanized by perforating the diaphragm while under deep anesthesia (sodium pentobarbital (Sanofi, Belgium), 75 mg kg^−1^, i.p.). The thoracic aorta was carefully removed and stripped of adherent tissue. Starting approximately 2 mm distally from the aortic arch, the descending aorta was cut into four segments of 2 mm length and immersed in Krebs Ringer (KR) solution (37°C, 95% O_2_/5% CO_2_, pH 7.4) containing (in mmol/L): NaCl 118, KCl 4.7, CaCl_2_ 2.5, KH_2_PO_4_ 1.2, MgSO_4_ 1.2, NaHCO_3_ 25, CaEDTA 0.025, and glucose 11.1. To assess the contribution of basal VSMC Ca^2+^ influx to the pressure‐dependency of aortic stiffness, Ca^2+^ was omitted from the KR solution and 1 mmol/L EGTA was added. To avoid any vasomotor interference due to prostanoids, 10 *μ*mol/L indomethacin (Federa, Belgium) was added to all experiments.

### Angiotensin‐II treatment

Mice (~6‐month‐old male C57BL/6JRj mice, *n* = 19 (Janvier Labs)) were randomly divided into experimental groups and treated either with saline (mean body weight: 32.6 ± 1.3 g, n = 9) or with angiotensin‐II (ang‐II) (mean body weight 32.3 ± 1.6 g, *n* = 10) via subcutaneous osmotic minipumps (model 1007D, Alzet, Cupertino, CA) for 7 days (1000 ng kg^−1^ min^−1^), as described previously (Leloup et al. 2018). Mice were anesthetized using sevoflurane (4–5% in O_2_, 1 L min^−1^) and placed onto a heating pad. Using a hemostat, a subcutaneous pocket wide enough for an osmotic minipump was created. The pocket was flushed with saline and the osmotic minipump was inserted. After closing the incision with sterile sutures, a single dose of 7.5 mg kg^−1^ ketorolac tromethamine (Ketalar^®^, Pfizer) was subcutaneously administered. Calculations of the required ang‐II concentrations were made per individual mouse based on its weight and by using the online tool provided by Alzet. After 7 days, mice were anesthetized using isoflurane (1.5% in 1 L min^−1^ O_2_), the descending thoracic aorta was removed and stripped of adherent tissue and 2 mm segments were immersed in Krebs Ringer solution, as described earlier in the materials and methods.

### ROTSAC

The aortic segment was mounted between two parallel wire hooks in 8 mL organ baths. Diameter and estimates of transmural pressure were derived as described previously (Leloup et al. 2016). In short, force and displacement of the upper hook were measured with a force‐length transducer connected to a data acquisition system (Powerlab 8/30 and LabChart 7, ADInstruments). Force and displacement were acquired at 1 kHz. To estimate the transmural pressure that would exist in the equilibrated vessel segment with the given distension force and dimensions, the Laplace relationship was used:

$$P=\frac{F}{l.D}$$

with *F* being the force, *l* the segment length (~2 mm) and *D* the diameter of the vessel segment. Force was measured directly by the transducer. The diameter of the vessel segment at a given preload was derived from the displacement of the upper hook, being directly proportional to the inner circumference:

$$D=\frac{2d}{\pi }$$

with d being the outer distance between the hooks (to approximate the inner circumference of the vessel segment). Before each experiment, diameter and length were determined at three different preloads (20, 40, and 60 mN) using a stereomicroscope and calibrated image software. To correct for the decrease in segment length with increased diameter, the average length of the segment at each cycle (100 msec) was derived from the diameter‐length relationship using basic linear regression.

The preload was adjusted until the desired diastolic and systolic pressure. At all pressures, stretch amplitude corresponding to 40 mmHg was chosen to allow calculation of compliance and Peterson modulus. Compliance (C) was calculated as follows:

$$C=\frac{\Delta D}{\Delta P}$$

with ∆*D* being the difference between systolic and diastolic diameter and ∆*P* being the pressure difference. The Peterson modulus of elasticity (*E*_p_) is a frequently used, vessel size‐independent measure of arterial stiffness (Gosling and Budge 2003) and was calculated as follows:

$$E_{p}=D_{0}.\frac{\Delta P}{\Delta D}$$

with *D*_0_ being the diastolic diameter. During all experiments, the segments were continuously stretched directly after mounting them in the organ bath with a frequency of 10 Hz to mimic the physiological heart rate in mice (600 bpm) and at physiological pressure (~80–120 mmHg). At approximately 60 min after isolation of the aorta from the animal, VSMCs were stimulated using the *α*_1_‐adrenergic agonist phenylephrine (PE) (Sigma‐Aldrich, Belgium). NΩ‐nitro‐l‐arginine methyl ester (L‐NAME) (Sigma‐Aldrich, Belgium) was used to inhibit endothelial nitric oxide synthase (eNOS). All measurements were performed from low pressure (40–80 mmHg or 60–100 mmHg for ang‐II experiments) to high pressure (180–220 mmHg or 220–260 mmHg for ang‐II experiments), stretch amplitude was 40 mmHg at all pressures. It took approximately 5–10 min to acquire measurements over the entire pressure range. Therefore, the measurements were done on steady‐state contractions, 30 min after the addition of the compound. The concentration‐response data were acquired by pre‐contracting four different segments in four parallel set‐ups with eight different concentrations of PE, hence every segment received two different doses of PE, with the lowest dose first. The organ baths were thoroughly flushed with fresh KR solution to wash away all PE and the measurements were repeated in the presence of 300 *μ*mol/L L‐NAME. All measurements were done on steady state contractions, 30 min after the addition of PE to the organ bath. The measurements in Ca^2+^‐free KR solution were done ~3 min after the switch to Ca^2+^ free KR solution as this interval is known to be sufficient to ensure complete depletion and chelation of free Ca^2+^ from the extracellular space (data not shown).

### Statistical analyses

All results are expressed as the mean ± SD with n representing the number of mice and analyses were performed using Prism 6.0 (GraphPad Software, La Jolla, CA). The effects of VSMC contraction or pressure on the measured vessel parameters were assessed using a two‐way ANOVA with repeated measures, if appropriate. A Bonferroni post hoc test was used to correct for multiple comparisons. Dose‐response curves were fitted with sigmoid concentration–response equations with variable slope, which revealed Emax‐ and logEC_50_‐values. A 5% level of significance was selected.

## Results

### Contribution of basal VSMC tone to the pressure‐dependency of aortic stiffness ex vivo

**Figure 1:**
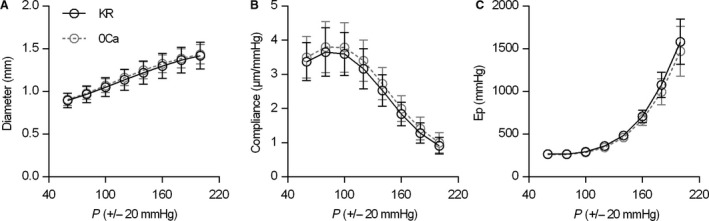
Contribution of basal VSMC tone to the pressure‐dependency of diastolic diameter (A), compliance (B), and *E*p (C). The *x*‐axis represents the average pressure at which the continuously oscillating vessel (with a stretch amplitude of 40 mmHg), was studied. Removal of extracellular Ca^2+^ (0Ca) from the Krebs‐Ringer (KR) solution did not significantly affect the pressure‐dependency of any of these parameters. Symbols and error bars represent the mean and 95% confidence intervals, respectively

The pressure‐dependency of diastolic diameter (D_0_), compliance and *E*p from an average distension pressure of 60 mmHg (40–80 mmHg) to an average distension pressure of 200 mmHg (180–220 mmHg), in the presence or absence of extracellular Ca^2+^, is shown in Figure 1. In the absence of any contractile agent, the presence of extracellular Ca^2+^ did not significantly affect the pressure‐dependency of aortic diameter (Fig. 1A), compliance (Fig. 1B) or *E*p (Fig. 1C), indicating that – in the absence of vasoactive compounds – the contribution of basal VSMC tone to the isobaric biomechanical properties of the healthy aorta was limited. Therefore, in the subsequent experiments, all baseline measurements (i.e., no VSMC activation) were performed in KR solution.

### Basal NO production is a crucial factor in the pressure‐dependency of aortic biomechanics

**Figure 2:**
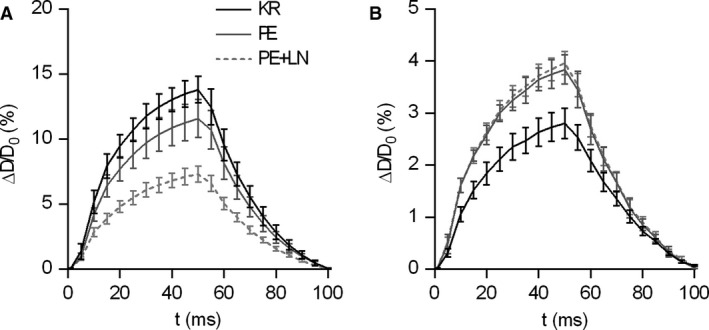
Average diameter distension of one cycle (100 msec) of the oscillating thoracic aorta segments (*n* = 5–6) at normal (80–120 mmgH, A) and high (180–220 mmHg, B) pressure. VSMC stimulation using either 1 μmol/L phenylephrine (PE) or PE in the presence of 300 μmol/L L‐NAME (PE+LN) decreased isobaric diameter distension as compared to the unstimulated distention in Krebs‐Ringer solution (KR) at normal pressure (80–120 mmHg), while pre‐contraction of the VSMCs increased isobaric diameter distension at high pressure (180–220 mmHg), independently from L‐NAME. Tracings were downsampled to 5 msec intervals. Line and error bars represent the mean and 95% confidence intervals.

We previously reported that activation of VSMCs increases isobaric stiffness in aortic segments of the mouse (Leloup et al. 2016). To illustrate the effects of VSMC contraction on the intrinsic biomechanics of the isolated aortic segments, the segments were maximally stimulated using 1 *μ*mol/L PE with or without 300 *μ*mol/L of the eNOS inhibitor L‐NAME. At normal pressure (80–120 mmHg), addition of 1 *μ*mol/L PE decreased the relative diameter change for the same pressure increment of 40 mmHg. Addition of 300 *μ*mol/L L‐NAME to inhibit basal NO production decreased relative distension, confirming the abundance of basal NO production in the aortic ECs (Fig. 2A). A stepwise increase in the distension force to high pressure (180–220 mmHg) was accompanied by a large reduction in relative diameter change, which was a direct consequence of the engagement of collagen fibers acting as load‐bearing components in the arterial wall (Fig. 2B). Interestingly, PE significantly attenuated the pressure‐associated decrease in distension, that is distension at 180–220 mmHg was larger when the VSMCs were pre‐contracted (Fig. 2B). Blocking of eNOS with L‐NAME did not affect distension at 180–220 mmHg, indicating that basal NO was ineffective at suppressing the effects of PE on aortic diameter distension in this pressure range.

**Figure 3:**
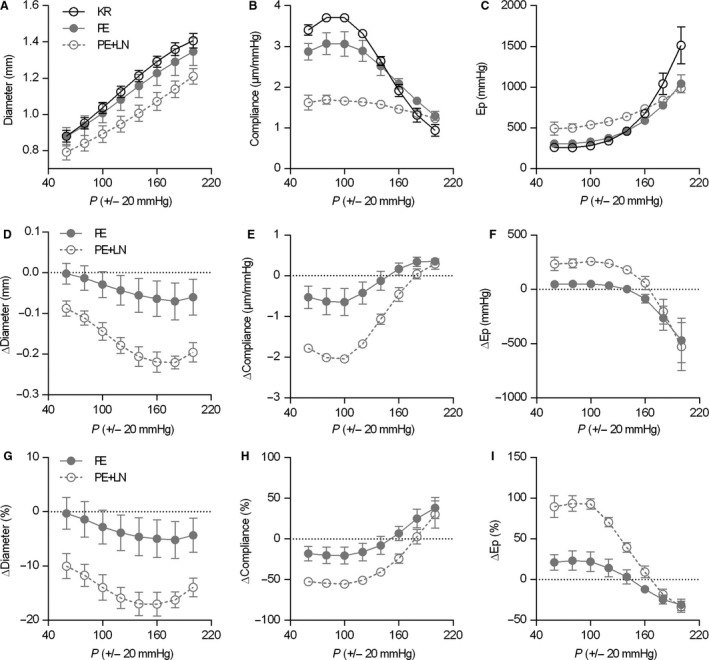
Pressure‐dependency of diastolic diameter (A), compliance (B), and *E*p (C) in unstimulated conditions (KR) and after stimulation with 1 μmol/L phenylephrine (PE), in the absence or presence of 300 μmol/L L‐NAME (LN). Panels from the first, second, and third row represent the absolute values (A–C), the isobaric change from baseline (KR) (D–F) and the relative isobaric change from baseline (KR) (G–I), respectively. Symbols and error bars represent the mean and 95% confidence intervals, respectively. For clarity, symbols describing the level of significance were omitted from this figure and can be found in Table S1.

The effects of VSMC and EC stimulation on the pressure‐dependency of aortic diameter and biomechanics at an extended pressure range from 60 mmHg (40–80 mmHg) to 200 mmHg (180–220 mmHg) are shown in Figure 3. The upper row shows the absolute pressure‐dependency of diastolic diameter (Fig. 3A), compliance (Fig. 3B) and *E*p (Fig. 3C), while the bottom two rows show the change in diameter, compliance and *E*p as compared to the relaxed segment (KR), in absolute (Fig. 3D–F) or relative (Fig. 2G–I) terms. VSMC contraction with 1 *μ*mol/L PE shifted the pressure‐diameter curve to smaller diameters over the entire pressure range (Fig. 3A, D, G). Compliance (Fig. 3B, E, H) increased and *E*p (Fig. 3C, F, I) decreased, but only at pressures below ~150 mmHg. At higher pressures, the direction of the effect changed, that is VSMC activation with PE limited the pressure‐dependent decrease in compliance and pressure‐dependent increase of *E*p.

To assess the contribution of basal NO, we added 300 *μ*mol/L L‐NAME to the PE‐containing KR solution. At all pressures, the addition of L‐NAME to the pre‐contracted segments resulted in a shift of the pressure‐diameter curve to lower diameters (Fig. 3A, D, G) and a shift of the compliance‐pressure curve to lower compliance at nearly all but the highest pressures (Fig. 3B, E, H). The pressure‐*E*p relationship shifted to higher stiffness values over nearly the entire pressure range (Fig. 3C, F, I), increasing *E*p at physiological pressure up to nearly 100% relative to unstimulated conditions (Fig. 3I), confirming earlier observations that VSMC tone plays an important role in determining the intrinsic biomechanical properties of the aortic wall. An overview of these values can be found in Table S1.

### Pressure‐dependence of the PE‐sensitivity of biomechanical properties

**Figure 4:**
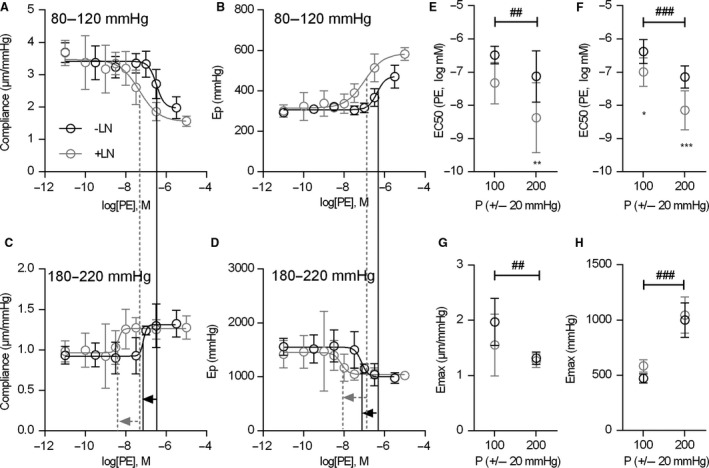
Dose response curves showing the compliance (A,C) and *E*p (B,D) response to pre‐contraction with PE at different concentrations at normal pressure (80–120 mmHg) and high pressure (180–220 mmHg) in the absence (‐LN, black) or presence (+LN, gray) of 300 μmol/L L‐NAME. The pressure‐dependent shifts in PE sensitivity in the absence and presence of L‐NAME are indicated with solid black and dashed gray lines and arrows, respectively. Dose‐response curves were fitted and EC50 (E, F) and Emax (G, H) values for compliance (E, G) and *E*p (F, H) were calculated. Two‐way ANOVA with Bonferroni post‐hoc test, *,**,*** *P* < 0.05, 0.01, 0.001 versus ‐LN. ##, ### *P* < 0.01, *P* < 0.001 for the pressure factor. Symbols and error bars represent the mean and 95% confidence intervals.

To date, only the isobaric effects of maximal α1‐adrenergic stimulation have been investigated. To increase our understanding of the physiological relevance of this experimental model, we assessed the sensitivity by which α1‐adrenergic stimulation affected the biomechanics at different pressures by pre‐contracting the segments with different concentrations of PE, in the absence or presence of L‐NAME. The effects on compliance (Fig. 4A, C) and *E*p (Fig. 4B, D) were plotted against the PE concentration to obtain concentration‐response curves at normal pressure (80–120 mmHg) and high pressure (180–220 mmHg). Fitted EC_50_ values for compliance (Fig. 4E) and *E*p (Fig. 4F) revealed that high pressure significantly shifted the PE sensitivity to lower concentrations both in the absence and presence of L‐NAME. Fitted E_max_ values for both compliance (Fig. 4G) and *E*p (Fig. 4H) showed a trend similar to the previous observations, that is basal NO supressed PE‐induced stiffening at normal pressure while it did not affect the maximal “de‐stiffening” effect of PE at high pressure. However, the effect of L‐NAME on fitted E_max_ values was not statistically significant, possibly due to the duration of the protocol and the associated time‐dependent loss of basal NO.

### Assessing the pressure‐dependency of arterial biomechanics in a mouse model of arterial disease

**Figure 5:**
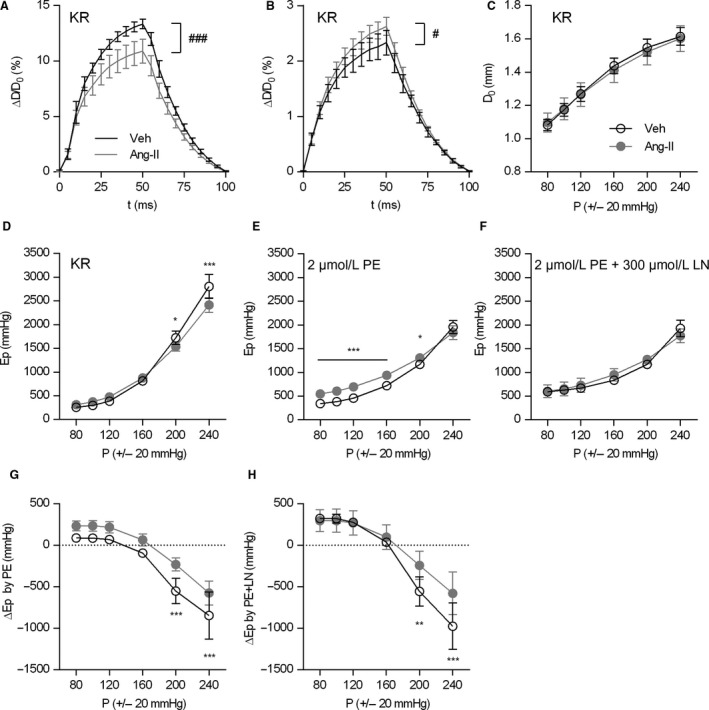
Pressure‐dependency of isobaric aortic stiffness in an angII‐mouse model. Maximal aortic distension at 80–120 mmHg (A) and 180–220 mmHg (B) was significantly different between 1‐week ang‐II‐treated (Ang‐II) and vehicle‐treated (Veh) mice. The pressure‐dependency of the diastolic diameter was not significantly different between groups in unstimulated conditions (C). Pressure‐*E*p relationship in Krebs‐Ringer (KR), (D) or after pre‐contraction with 2 μmol/L phenylephrine (PE) in the absence (E) or presence (F) of 300 μmol/L L‐NAME (LN) to block basal NO production. The isobaric change in *E*p (vs. KR) with PE or PE+LN is shown in (G) and (H), respectively. Tracings in (A, B) are downsampled to 5 msec intervals. Two‐way ANOVA with Bonferroni post‐hoc test for multiple comparisons. #, ### *P* < 0.05, 0.001 for the treatment factor in (A, B). *,**,*** *P* < 0.05, 0.01, 0.001 versus vehicle. Symbols and error bars represent the mean and 95% confidence intervals.

To assess the (patho)physiological relevance of this concept, mice were treated with ang‐II for 1 week. The ang‐II‐treated mice display increased basal VSMC tone and compromised basal NO signaling after 1 week of treatment, as described previously (Leloup et al. 2018). To assess how the pressure‐dependency of isobaric stiffness was affected by 1‐week of ang‐II treatment, the pressure‐*E*p relationship was analyzed at a broad pressure range (up to 220–260 mmHg) and at different levels of VSMC stimulation (Fig. 5). Tracings of the diameter distension showed decreased maximal distension at 80–120 mmHg (Fig. 5A) while the maximal distension was increased in ang‐II‐treated mice, compared to controls (Fig. 5B). The diastolic diameter in unstimulated (KR) conditions was not different for the entire pressure range (Fig. 5C), but plotting *E*p against pressure revealed a trend toward increased stiffness at pressures below ~160 mmHg and decreased *E*p at pressures above ~160 mmHg (Fig. 5D), suggesting increased basal VSMC tone in ang‐II‐treated mice. Stimulation of the aortic segments with 2 *μ*mol/L PE (Fig. 5E) shifted the *E*p‐pressure relationship below ~160 mmHg more upwards in ang‐II‐treated animals, compared to controls (Fig. 5G). At the same time, the PE‐associated decrease in isobaric *E*p at high pressures was smaller in ang‐II‐treated animals and also occurred at higher pressures. Addition of 300 *μ*mol/L L‐NAME to the organ bath (Fig. 5F) shifted the pressure‐*E*p curve of the control animals to the curve of the ang‐II‐treated animals, confirming our previous observations that basal NO efficacy was affected in the ang‐II‐treated animals.

## Discussion

We assessed the interactions between VSMC tone, distending pressure and isobaric biomechanics of isolated mouse aortic segments. Previous studies determining the effects of VSMC contraction and relaxation on large artery stiffness have reported conflicting results (Peterson et al. 1960; Dobrin and Rovick 1969; Aars 1971; Cox 1976; Yano et al. 1989; Armentano et al. 1990; Barra et al. 1993; Bank et al. 1995, 1996; Chamiot‐Clerc et al. 1998; Leloup et al. 2016), which is – at least partly – caused by the large variance in experimental conditions and methodologies used, including the use of an in vivo or ex vivo experimental design, the former typically involving an isobaric, dynamic assessment, the latter often involving an isometric, static approach. We used an in‐house developed ex vivo set‐up to apply high frequency cyclic stretch, to minimalize the time‐dependent loss of basal NO efficacy associated with the shift of the vessel from an in vivo to an ex vivo environment (van Langen et al. 2012; Leloup et al. 2017). In fact, the time‐dependent decrease in basal NO efficacy could be contributing to some of the discrepancies observed between dynamic or in vivo and static or ex vivo properties of large artery biomechanics that have been reported in literature. This includes one study where the ratio of static and dynamic canine carotid artery stiffness was observed to be as high as 2.5 (Gow and Hadfield 1979) and another study that showed that infusion of vasoactive drugs affected the pressure‐dependency of large artery stiffness only moderately in vivo, while it had large effects ex vivo (Butlin et al. 2015). The ex vivo approach in this study allowed precise control of the extracellular environment and eliminated the indirect effects of PE or L‐NAME when infused systemically (i.e., via blood pressure or heart rate) on aortic biomechanics. This allowed us to investigate the independent contributions of VSMC tone and distension pressure on the intrinsic biomechanical properties of the aorta.

We found that basal Ca^2+^ influx – which typically contributes slightly to isometric preload of isolated aortic rings (De Moudt et al. 2017) – did not significantly contribute to the isobaric properties of the aortic rings in this set‐up. However, VSMC stimulation with PE affected isobaric stiffness differently at low versus high pressure. At pressures below ~150 mmHg, VSMC contraction with PE increased *E*p and decreased compliance. However, several observations indicate that aortic VSMCs and ECs are functionally adapted to limit the stiffening‐effects of PE in this pressure range. We hypothesize that – in the healthy aorta – the large amount of basal NO production by the endothelial cells and the relatively low sensitivity of the VSMC to PE itself suppress the stiffening‐effects of PE in this pressure‐range, thereby protecting the cardiovascular system from unfavorable hemodynamic conditions. The circulating catecholamine concentrations in blood are strongly dependent on the physiological state of the organism and can rise rapidly with several orders of magnitude during physiological stress. The basal circulatory catecholamine levels in mice correspond roughly to 3‐5 × 10^−8^ M PE (Russell and Watts 2000; Grouzmann et al. 2003). These concentrations did not affect the biomechanical properties of the isolated aortic segment at normal resting blood pressure, as long as the endothelium was functionally intact. Blocking eNOS with L‐NAME resulted in a significant decrease in isobaric compliance and an increase in *E*p at physiological catecholamine concentrations and physiological distension pressures. This confirms experimental studies on humans showing that local basal NO inhibition in the brachial (Kinlay et al. 2001) or iliac (Schmitt et al. 2005) artery indeed increases the stiffness of this vessel. On the other hand, the direction of the effect of PE on isobaric compliance and *E*p switched at pressures above ~150 mmHg. The sensitivity of the VSMCs to the de‐stiffening effects of PE‐induced contraction was increased, compared to lower pressures and basal NO signaling was virtually ineffective in suppressing the de‐stiffening effects of PE. These results suggest that the VSMCs and ECs are physiologically adapted to suppress the stiffening‐effects of VSMC contraction, while facilitating the de‐stiffening effects of VSMC contraction at high pressure. The dependency of PE‐sensitivity on distension pressure observed in this study corresponds well with a previous study on the isometric contraction properties at different preloads. Indeed, increasing preload (i.e., static distension) was found to increase the sensitivity to PE, while the effect of L‐NAME on the maximal contraction with PE (i.e., basal NO efficacy) decreased with isometric preload (De Moudt et al. 2017).

The potential mechanisms by which VSMC tone affects the aortic stiffness‐pressure relationship could be explained by considering the modified Maxwell model of the brachial artery wall, proposed by A. Bank and colleagues (Bank et al. 1996). The model, of which a schematic representation can be found in the original publication, describes the presence of elastin and collagen in parallel with the VSMC, and a collagen component in series with the VSMCs. At low pressure, when elastin is the main load‐bearing component of the aortic wall, VSMC contraction will increase stiffness by engaging a series of collagen fibers. Recent observations also show that actin‐myosin interactions alter VSMC cell stiffness structurally as well as functionally (Zhu et al. 2012; Saphirstein et al. 2013; Gao et al. 2014; Hong et al. 2015), and altered intrinsic VSMC stiffness has been observed in animal models of increased arterial stiffness (Sehgel et al. 2015). This suggests that increased intrinsic VSMC rigidity also contributes to decreased isobaric compliance of the contracted aortic wall, independent from the shift of wall stress from elastin to series collagen. When aortic distension pressure increases, VSMC contraction will disengage parallel collagen fibers, thereby shifting wall stress from the stiff collagen fibers to the VSMCs and elastin fibers. The increase in compliance or decrease of *E*p with VSMC contraction is only present at high pressure because parallel collagen is only engaged at high strain. In addition, there is a geometric component that acts, especially at high pressure, via the same principle. VSMC contraction will shift the aorta to a smaller isobaric diameter, thereby automatically reducing the relative contribution of parallel collagen as the load‐bearing component of the aortic wall. In fact, comparing strain‐matched vessels with different levels of VSMC tone thus involves the comparison of vessels at different transmural pressures, rendering the interpretation of the physiological significance difficult. The geometric contribution to VSMC tone‐associated changes in isobaric wall strain is likely responsible for the differences in the effects of isometric and isobaric VSMC contraction on large artery compliance reported in literature (Barra et al. 1993).

We assessed the pathophysiological relevance of this concept in the ang‐II‐treated mouse, a well‐known model of arterial disease. This model showed reduced basal NO efficacy and increased VSMC tone and – in line with the ex vivo effects of VSMC tone on isobaric stiffness – isobaric stiffness at physiological BP was increased, while the pressure‐associated increase of stiffness at high pressures was reduced, compared to the controls. Upon VSMC stimulation with PE, the segments of the ang‐II‐treated mice demonstrated a reduced capacity for modulating the pressure‐stiffness relationship, suggesting that not only increased isobaric stiffness at normal pressure, but also a reduced capacity of the VSMCs to limit the pressure‐associated increase in aortic stiffness, which may contribute to the pathogenesis of this mouse model. It remains to be elucidated how this contributes to arterial disease in humans and whether pharmacological interventions that affect VSMC contractility – also in the large arteries – affect the pressure‐stiffness relationship and, hence, overall regulation of large artery hemodynamics.

We previously described the advantages of the ROTSAC set‐up, including stretching the vessels at high frequency while accurately controlling the extracellular environment and distension force. Even though this allowed us to determine the independent contributions of EC‐VSMC physiology and distension pressure on isobaric biomechanics, it is difficult to translate this directly to the in vivo setting without considering the effects of isolating the vessel from the organism. Firstly, the lack of flow potentially influences shear stress‐dependent pathways in the endothelial cells (Pohl et al. 1986; Davies 1995; Corson et al. 1996). However, a review of the literature of shear stress measurements in healthy volunteers revealed that shear stress is lower in the aorta versus other parts of the arterial tree (Pantos et al. 2007), indicating that the contribution of shear stress is more important in the muscular arteries for regulating vessel diameter and, hence, flow, while the elastic arteries – where cyclic deformation is larger – rely more on stretch‐dependent basal NO. Indeed, cyclic stretch affects the endothelial cells both on a molecular (Peng et al. 2003) and functional (Leloup et al. 2017) level. Secondly, upon isolating the aorta, neurogenic and hormonal stimuli are lost and these have been shown to play a role in regulating large artery mechanics (Pagani et al. 1975; Barra et al. 1993). A study that compared the effects of vasoactive substances on the aorta found large differences in vivo versus ex vivo (Butlin et al. 2015) and visco‐elastic properties of the aorta change significantly after isolation of the aorta from the animal (Boutouyrie et al. 1997). This may partly be explained by the time‐dependent reduction in basal NO efficacy ex vivo versus in vivo. Thirdly, the protocol used in this study measured the effects of VSMC activation on the acute pressure‐dependent changes in aortic biomechanics. As reported above, all measurements were done in a relatively short time‐period, on steady‐state contractions and starting at low pressure. The visco‐elastic nature of the aortic wall as well as the mechanosensitivity of the VSMC‐EC axis (Leloup et al. 2017) suggest that the effects seen at high pressure are time‐dependent. Further research is needed to elucidate how extended periods of increased (pulse) pressure affect the interaction between VSMC‐EC cross‐talk and aortic structure, but this is beyond the scope of this study.

Even though one should be cautious when translating observations from this set‐up to the physiological in vivo setting, several studies provide indications that VSMC‐tone indeed plays an important role in the regulation of aortic stiffness in vivo. A study on the pressure‐dependency of aortic stiffness in humans showed that there is significant hysteresis between systolic BP and pulse transit time – as a measure of arterial stiffness – indicating that the large artery blood pressure‐stiffness relationship is acutely affected by other factors than blood pressure alone (Liu et al. 2014). Also, a study on the acute effects of smoking on arterial stiffness showed that smoking resulted in an acute increase in arterial stiffness through contribution of pressure‐independent factors (Stefanadis et al. 1998), hypothesized by the authors to be autonomic nervous system or endothelium‐related factors. Another study on the circadian changes in arterial stiffness showed a paradoxical increase in arterial stiffness at night, while BP dropped, serving as another indication that the active changes in large artery wall properties can modulate large artery biomechanics (Bia et al. 2008), aside from the passive changes due to blood pressure alone.

In conclusion, the overall aim of this study was to acquire a physiologically relevant experimental model of the individual contribution of VSMC tone and distension pressure to the isobaric biomechanical properties of mouse aorta segments. We found that the unique property of the large, elastic arteries to produce large amounts of basal NO, combined with a pressure‐dependent α1‐adrenergic response, translates into an elegant concept that ensures high aortic compliance in the physiological pressure range and facilitates de‐stiffening effects of VSMC contraction at high pressure. Further research is needed to understand how this concept integrates into the complex interplay between heart, arteries, and periphery, how it contributes to the regulation of large artery hemodynamics with changing blood pressure and circulating catecholamines, as well as the hemodynamic consequences of pharmaceutical modulation of VSMC tone and blood pressure in arterial disease.

## Conflict of Interest

None.

## Supporting information


**Table S1:** The effects of VSMC activation and eNOS blockade on the geometrical and the isobaric biomechanical properties of isolated aortic segments.
