# Supplementary material for: Vascular smooth muscle cell contraction and relaxation in the isolated aorta: a critical regulator of large artery compliance
Source: Physiol Rep. 2019 Feb 27;7(4):e13934. doi: 10.14814/phy2.13934 (PMC6391714; doi:10.14814/phy2.13934)
Supplement: Supplementary file 1 — Table S1: The effects of VSMC activation and eNOS blockade on the geometrical and the isobaric biomechanical properties of isolated aortic segments. [file PHY2-7-e13934-s001.pdf]

Supplementary Table S1: The effects of VSMC activation and eNOS blockade on the geometrical and the isobaric biomechanical properties of isolated aortic segments.

		D_0 (mm)			Compliance (μm/mmHg)			Ep (mmHg)		
	⟨P⟩ (mmHg)	KR	PE	PE+LN	KR	PE	PE+LN	KR	PE	PE+LN
Absolute values	60	0.88 (0.03)	0.88 (0.04) [1]	0.79 (0.03) [3, c]	3.40 (0.10)	2.87 (0.16) [3]	1.62 (0.15) [3, c]	259 (15)	305 (11)	493 (65) [3, c]
	80	0.95 (0.03)	0.94 (0.04) [3]	0.84 (0.03) [3, c]	3.71 (0.05)	3.07 (0.22) [3]	1.69 (0.09) [3, c]	257 (10)	306 (11)	497 (42) [3, c]
	100	1.04 (0.02)	1.01 (0.04) [3]	0.89 (0.04) [3, c]	3.70 (0.04)	3.06 (0.24) [3]	1.66 (0.05) [3, c]	280 (9)	330 (16)	537 (32) [3, c]
	120	1.12 (0.03)	1.08 (0.05) [3]	0.95 (0.04) [3, c]	3.31 (0.04)	2.89 (0.21) [3]	1.64 (0.04) [3, c]	339 (7)	375 (13)	578 (24) [3, c]
	140	1.21 (0.03	1.16 (0.05) [3]	1.01 (0.04) [3, c]	2.64 (0.09)	2.52 (0.18) [1]	1.58 (0.05) [3, c]	459 (12)	460 (16)	638 (19) [3, c]
	160	1.29 (0.02)	1.23 (0.05) [3]	1.07 (0.04) [3, c]	1.92 (0.12)	2.09 (0.10) [2]	1.46 (0.03) [3, c]	675 (38)	588 (10) [2]	733 (19) [c]
	180	1.36 (0.03)	1.29 (0.06) [3]	1.14 (0.04) [3, c]	1.32 (0.14)	1.66 (0.07) [3]	1.36 (0.07) [c]	1042 (105)	776 (39) [3]	837 (34) [3]
	200	1.41 (0.03)	1.35 (0.06) [3]	1.21 (0.03) [3, c]	0.94 (0.12)	1.30 (0.09) [3]	1.23 (0.04) [c]	1515 (182)	1044 (89) [3]	986 (23) [3]
			ΔD_0 (mm)			ΔCompliance (μm/mmHg)			ΔEp (mmHg)	
			PE	PE+LN		PE	PE+LN		PE	PE+LN
Absolute isobaric change *versus* KR	60		-0.00 (0.02)	-0.09 (0.02) [c]		-0.53 (0.22)	-1.78 (0.08) [c]		47 (14)	234 (50) [c]
	80		-0.01 (0.02)	-0.11 (0.01) [c]		-0.63 (0.26)	-2.01 (0.05) [c]		49 (17)	241 (33) [c]
	100		-0.03 (0.03)	-0.14 (0.02) [c]		-0.64 (0.27)	-2.04 (0.06) [c]		50 (20)	258 (24) [c]
	120		-0.04 (0.03)	-0.18 (0.02) [c]		-0.42 (0.22)	-1.68 (0.08) [c]		35 (18)	238 (21) [c]
	140		-0.06 (0.03)	-0.21 (0.02) [c]		-0.13 (0.19)	-1.07 (0.10) [c]		1 (23)	179 (25) [c]
	160		-0.06 (0.04)	-0.22 (0.01) [c]		0.17 (0.12)	-0.46 (0.13) [c]		-87 (32)	58 (49) [c]
	180		-0.07 (0.04)	-0.22 (0.01) [c]		0.35 (0.09)	0.05 (0.10) [c]		-265 (90)	-204 (92) [a]
	200		-0.06 (0.04)	-0.20 (0.02) [c]		0.36 (0.08)	0.29 (0.10)		-471 (165)	-529 (178) [a]
			ΔD_0 (%)			ΔCompliance (%)			ΔEp (%)	
			PE	PE+LN		PE	PE+LN		PE	PE+LN
Relative isobaric change *versus* KR	60		-0.3 (2.4)	-10.0 (1.9) [c]		-15.4 (6.2)	-52.4 (3.2) [c]		18.3 (6.4)	89.9 (14.1) [c]
	80		-1.4 (2.6)	-11.7 (1.6) [c]		-17.1 (6.7)	-54.3 (1.8) [c]		19.3 (7.1)	93.5 (10.2) [c]
	100		-2.8 (2.5)	-14.0 (1.9) [c]		-17.4 (7.0)	-55.2 (1.3) [c]		18.1 (7.5)	92.0 (6.6) [c]
	120		-3.9 (2.7)	-15.9 (1.6) [c]		-12.7 (6.5)	-50.6 (1.7) [c]		10.5 (5.6)	70.2 (6.1) [c]
	140		-4.6 (2.8)	-17.0 (1.7) [c]		-4.7 (7.0)	-40.3 (2.8) [c]		0.4 (5.1)	39.1 (6.3) [c]
	160		-5.0 (2.8)	-17.0 (1.8) [c]		9.0 (6.7)	-23.6 (5.7) [c]		-12.7 (4.0)	9.0 (7.5) [c]
	180		-5.2 (2.8)	-16.3 (1.1) [c]		27.1 (10.6)	4.1 (8.6) [c]		-25.1 (5.7)	-19.1 (6.5)
	200		-4.3 (2.6)	-13.9 (1.4) [c]		38.9 (13.6)	32.3 (16.6)		-30.6 (7.3)	-34.3 (7.1)

Values are shown as mean (SD) with n=5 for all conditions. Repeated measures two-way ANOVA with Bonferroni post-hoc test for multiple comparisons
[1.2.3]: $P<0.05$. $P<0.01$. $P<0.001$ vs. KR and [a. b. c]: $P<0.05$. $P<0.01$. $P<0.001$ vs PE/ΔPE. KR: Krebs-Ringer, PE: 1 μM phenylephrine; LN: 300 μM L-NAME
